# A two-arm, randomised feasibility trial using link workers to improve dental visiting in people with severe mental illness: a protocol paper

**DOI:** 10.1186/s40814-023-01383-2

**Published:** 2023-09-08

**Authors:** Claire Hilton, Abigail Morris, Girvan Burnside, Rebecca Harris, Vishal R. Aggarwal, Sarah Procter, Robert Griffiths, Paul French, Louise Laverty, Fiona Lobban, Katherine Berry, David Shiers, Rebecca Golby, Fanni Fazekas, Kyriakos Valemis, Antonia Perry, Connie Newens, Eirian Kerry, Pauline Mupinga, Efstathia Gkioni, Christopher Lodge, Alison Dawber, Emma Elliott, Farah Lunat, Jasper Palmier-Claus

**Affiliations:** 1 The Spectrum Centre for Mental Health Research, Lancaster University, Lancaster, UK; 2 Institute of Population Health, University of Liverpool, Liverpool, UK; 3 School of Dentistry, University of Leeds, Leeds, UK; 4 Lancashire & South Cumbria NHS Foundation Trust, Lancashire, UK; 5 Division of Psychology & Mental Health, University of Manchester, Manchester, UK; 6 Greater Manchester Mental Health NHS Foundation Trust, Manchester, UK; 7 Manchester Metropolitan University, Manchester, UK; 8 Pennine Care NHS Foundation Trust, Ashton-Under-Lyne, UK; 9 Division of Informatics, Imaging and Data Sciences, University of Manchester, Manchester, UK; 10 Liverpool Clinical Trials Centre, Clinical Directorate, University of Liverpool, Liverpool, UK

**Keywords:** Psychosis, Bipolar, SMI, Oral, Inequalities, Access, Dental

## Abstract

**Background:**

People with severe mental illness (e.g. psychosis, bipolar disorder) experience poor oral health compared to the general population as shown by more decayed, missing and filled teeth and a higher prevalence of periodontal disease. Attending dental services allows treatment of oral health problems and support for prevention. However, people with severe mental illness face multiple barriers to attending routine dental appointments and often struggle to access care. Link work interventions use non-clinical support staff to afford vulnerable populations the capacity, opportunity, and motivation to navigate use of services. The authors have co-developed with service users a link work intervention for supporting people with severe mental illness to access routine dental appointments. The Mouth Matters in Mental Health Study aims to explore the feasibility and acceptability of this intervention within the context of a feasibility randomised controlled trial (RCT) measuring outcomes related to the recruitment of participants, completion of assessments, and adherence to the intervention. The trial will closely monitor the safety of the intervention and trial procedures.

**Methods:**

A feasibility RCT with 1:1 allocation to two arms: treatment as usual (control) or treatment as usual plus a link work intervention (treatment). The intervention consists of six sessions with a link worker over 9 months. Participants will be adults with severe mental illness receiving clinical input from secondary care mental health service and who have not attended a planned dental appointment in the past 3 years. Assessments will take place at baseline and after 9 months. The target recruitment total is 84 participants from across three NHS Trusts. A subset of participants and key stakeholders will complete qualitative interviews to explore the acceptability of the intervention and trial procedures.

**Discussion:**

The link work intervention aims to improve dental access and reduce oral health inequalities in people with severe mental illness. There is a dearth of research relating to interventions that attempt to improve oral health outcomes in people with mental illness and the collected feasibility data will offer insights into this important area.

**Trial registration:**

The trial was preregistered on ISRCTN (ISRCTN13650779) and ClinicalTrials.gov (NCT05545228).

**Supplementary Information:**

The online version contains supplementary material available at 10.1186/s40814-023-01383-2.

## Background

The term severe mental illness refers to psychological difficulties that are debilitating and can adversely affect a person’s ability to function, and typically includes diagnoses of psychotic and affective disorders. Oral health in people with severe mental illness is poor compared to the general population [1–4]. This includes high rates of decayed, missing, and filled teeth [4], periodontal disease [5] and problematic levels of plaque [6]. Poor oral health can have a significant and wide-reaching impact on peoples’ lives affecting their self-esteem, life satisfaction, and daily functioning [7–9]. It can interrupt basic everyday functions like eating or drinking [10] and add to the already considerable personal burden of living with severe mental illness.

Multiple factors likely contribute to the poor oral health outcomes seen in people with severe mental illness [11]. People with severe mental illness are more likely to smoke [12], use recreational drugs [13], and have a poor diet [14], which can adversely affect their teeth and gums. They are also less likely to engage in oral health behaviours, such as flossing and brushing [15], which may be due to difficulties maintaining everyday activities during periods of relapse [16]. Mental health treatments may also have iatrogenic effects for oral health. For example, dry mouth is a side effect of many antipsychotic medications and can raise the risk of dental decay and soreness, which may interfere with chewing and swallowing [17, 18]. Unfortunately, hyposalivation can sometimes increase with greater numbers of medications taken meaning that the oral health of patients with the most severe psychiatric symptoms are most affected [19].

Dental services can prevent and treat oral health problems. However, a recent meta-analysis has suggested that people with severe mental illness are less likely to attend routine dental appointments [15]. One estimate suggests that only a third of people experiencing severe mental illness attended an annual dental appointment over a 3-year period [20]. A lack of perceived need and motivation, dental anxiety, practical barriers, and financial costs can all affect peoples’ likelihood of attending a dentist [21, 22]. Experiences such as paranoia and hallucinations can exacerbate anxiety around using dental services [16]. Addressing barriers to dental visiting and supporting people with severe mental illness to access routine preventive and therapeutic care may be an important step in improving their oral health.

Link work interventions aim to overcome practical and socio-economic barriers to help seeking that perpetuate and widen health inequalities around oral health [23]. Typically, in link work interventions, non-professional support workers assist vulnerable populations to navigate and bridge the gap between services. An initiative in Scotland, *Childsmile,* observed that vulnerable, marginalised families receiving link work were twice as likely to attend a dental practice [24]. Link work interventions have attempted to facilitate care pathways for people with Tuberculosis [25], screenings for diabetes [26], and engagement in activities for people with long-term health conditions [27]. Benefits to chronic health conditions have included improved illness management, resilience, and problem solving [28, 29]. Link work interventions have also supported people with mental health difficulties to access services [30, 31] and transition between primary and secondary care [29]. However, evaluations are typically small scale and there is a lack of randomised controlled trials of link work interventions. The authors are not aware of any evaluations of link work interventions to support people with severe mental ill health to access dental care.

The Mouth Matters in Mental Health Study is a feasibility randomised controlled trial (RCT) that aims to explore the feasibility, acceptability, and safety of a link work intervention to help people with severe mental illness currently receiving care from a community mental health service to access dental services. The intervention is the product of a collaboration between service users with severe mental illness, dental professionals, and mental health clinicians. Feasibility outcomes include the ability to recruit participants, gather outcome data, and retain people in the intervention. A qualitative evaluation will evaluate the acceptability and safety of the intervention and trial, alongside serious adverse events monitoring. This manuscript outlines the protocol used in the feasibility trial.

## Method

### Design

We will conduct a feasibility RCT with 1:1 allocation of participants to two arms: treatment as usual (TAU) or TAU plus the link work intervention. TAU will be whatever concomitant help the participant already has to facilitate dental attendance. Research staff will complete assessments at baseline and after 9 months. We will access routinely collected dental visiting data via the NHS Business Services Authority (NHSBSA). A qualitative evaluation of the intervention and trial will run concurrently with the feasibility trial. This protocol adheres to the Standard Protocol Items Recommendations for Interventional Trials (SPIRIT) statement [32]. Please see Supplementary Table S1.

### Randomisation

Randomisation will be via a randomisation programme managed by Liverpool Clinical Trials Centre (LCTC). This will allocate participants to a trial arm at a 1:1 ratio, stratified by the three sites (Lancashire Care NHS Foundation Trust; Pennine Care NHS Foundation Trust; Greater Manchester Mental Health NHS Foundation Trust). Allocation blocks will be random and concealed from trial staff.

### Blinding

Research staff conducting follow-up assessments will be blind to treatment allocation. Substantial efforts will be made to prevent unblinding, including regular reminders on blinding to staff, referrers and participants, and separate telephone numbers for link workers. Trial statisticians will be blind until the finalising of the statistical analysis plan. In some instances, it may be necessary to unblind staff in cases of immediate risk or emergencies. In cases of research assistants becoming unblinded, we will attempt to re-blind by using a second member of staff. We will monitor and report rates of unblinding in the final report.

### Participants

Participants will be people with severe mental illness accessing mental health services, but who have not attended a routine or planned dental appointment in the past 3 years. Routine and planned dental appointments will include any dental examination, diagnosis, advice, or treatment resulting from a routine appointment at a dental service. We will not consider emergency dental care (e.g. attendance at A&E) within this definition, although any follow-up routine and planned appointment would exclude the person from participating. The choice of 3 years without an appointment was based on the National Institute of Clinical Excellence (NICE) recall guidance [33] and is consistent with previous trials [34]. Participants will have to be aged 18 or older, able to provide informed consent and in receipt of care from a community mental health team (CMHT) or early intervention service for psychosis (EIS) at the point of referral. These services have been developed to work with severe and complex mental health issues. Eligibility is therefore based on service provision, rather than diagnostic criteria. However, we will record the diagnostic composition of the sample using the Mini International Neuropsychiatric Interview [35]. Exclusion criteria include inpatient status; immediate risk to self or others; and enrolment on another dental trial. The inclusion and exclusion criteria were set to be pragmatic, whilst ensuring the safety of participants and staff.

### Recruitment and consent

Recruitment will take place across three NHS Trusts in the Northwest of England. Research assistants, clinical studies officers, and assistant clinical research practitioners will inform CMHT and EIS staff about the research and share recruitment materials. CMHT and EIS staff can then discuss the research with people with severe mental illness accessing their service and, if they consent, refer them into the trial. We will also disseminate via recruitment posters placed in services and accept self-referrals by service users directly. All potential participants will have at least 24 h to consider whether to take part in the trial and complete a brief screening telephone or video call, or face-to-face visit, to ensure eligibility. Research assistants will meet participants at a place of mutual convenience to complete the written consent process, before conducting the baseline assessments. In cases where face-to-face meeting is difficult, to increase inclusivity, we will allow the option of audio recorded consent over telephone or video call. When appropriate, research assistants will be accompanied by interpreters to support participants’ understanding of the research and assessments.

### TAU and concomitant treatments

Given the inclusion criteria around receipt of service, participants will likely be receiving input from a mental health team, which often comprise of psychiatric nurses, social workers, support workers, occupation therapists, psychiatrists, psychological therapists, and clinical psychologists. Treatments often include medication, care coordination, and psychological therapy, focusing primarily on a person’s mental health. Sometimes these services offer monitoring and support around people’s physical health, which could include oral health, but this is rarely a primary focus. Participants will be able to access assessment, information, and treatment from dental services as normal. The control arm of this study will help to illuminate what constitutes TAU in dental care for people with severe mental illness.

### Treatment

The link work intervention aims to empower and support people with severe mental illness to access routine or planned dental appointments, whilst helping them to navigate the dental system and forge pathways to care. It is consistent with the COM-B model [36], which suggests that capability, opportunity, and motivation interact to facilitate behaviour change. This model acknowledges that intra-and external factors may limit behaviour. It suggests that the environment and its cultural milieu may affect opportunity; here, routine mental health care may not consider or prompt consideration of oral health and therefore opportunities for behaviour change are lost. The current intervention builds psychological and physical capacity around dental visiting (capability), including promoting demystification, knowledge exchange, anxiety management, and empathic social support (capability). It may involve helping people to book, plan and attend appointments through joint visiting/problem solving, apply for free/subsidised dental care, and advocate on behalf of patients (opportunity). Lastly, it may involve using simple motivational strategies to reinforce reasons for engaging with the process and seeing a dentist through motivational interviewing techniques (motivation).

Link workers will have up to six 1:1 sessions with participants over 9 months. They may assist people face-to-face or remotely by phone, telemedicine platforms, letter, email, or via staff/carers. Visits can occur at places of mutual convenience, such as people’s homes, clinics, or GP surgeries. Link workers can accompany patients to dental appointments facilitating transport if necessary. All link workers will receive training and supervision in basic behavioural change, motivation interviewing, and cognitive behavioural therapy informed techniques, and be equipped with knowledge of dental health provision and benefit applications. Link workers will share information about local services, what to expect at dental visits, costing models for dentistry, and available financial support. They will regularly monitor and share information on NHS dentists accepting patients. The treatment manual was developed through a series of co-design workshops with service users, dental staff, carers, and mental health professionals. Adherence to the manual will be monitored during the trial through use of sessional checklists and clinical supervision.

### Outcomes

#### Feasibility outcomes

This RCT is primarily concerned with feasibility outcomes. This includes recruitment of participants, completeness of outcome measures, and adherence to the intervention. As can be seen in Table 1, green outcomes would lead to progression to a full trial. One or more amber outcome would indicate that adaptions to the trial protocol and/or intervention manual would be required prior to a definitive trial. One or more red outcomes would mean that major alterations would be required before conducting a full trial. We will collect qualitative data on the acceptability of the trial procedures and intervention.

**Table 1:** Feasibility progression criteria employing traffic light indicators

Criterion	Critical feasibility outcome	Green	Amber	Red
(1) Recruitment rate	Feasibility of being able to recruit 84 participants within a 7-month window	≥ 80%	60–79%	≤ 59%
(2) Visiting Data	Percentage of participants with available data on dental visiting via self-report or BSA	≥ 80%	60–79%	≤ 59%
(3) Clinical exam	Percentage of participants completing the dental examination	≥ 80%	60–79%	≤ 59%
(4) Adherence to intervention	Percentage of participants receiving ≥ 1 intervention sessions during 9-month window	≥ 80%	60–79%	≤ 59%
(5) Intervention and trial protocol	Qualitative data to understand the acceptability and feasibility of the procedures, assessments, and intervention to inform a full trial and service delivery			
(6) Safety of intervention	Monitoring and review of research related serious adverse events (SAEs). The TSC will oversee SAEs across treatment arms. We will discontinue the trial if the intervention or procedures elevate risk			

#### Other outcomes

The proposed primary outcome for a definitive trial is planned or routine care appointments with a dental service. All participants will be asked ‘Have you attended a dental service since the baseline assessment (9 months ago)? This would include a routine appointment with a dentist or Special Care dentistry service. It could also include a planned appointment at a dental hospital. However, it would not include an emergency dental appointment’. Researchers will ask participants to confirm the nature and timing of the appointments (Table 2).

**Table 2:** Overview of outcomes by timepoint

Assessment	Baseline	9 month
Demographics and clinical information	X	X
Self-reported dental visiting		X
BSA recorded dental visiting		X
OHIP-14	X	X
Confidence in dental visiting item	X	X
MDAS	X	X
BPI-SF	X	X
MOPDH	X	X
RESES	X	X
PHQ-9	X	X
EQ-5D-5L	X	X
Access to free/subsidised dental care	X	X
Number of routine dental visits		X
Oral health hygiene behaviour items	X	X
MINI	X	-
Dental exam (optional) - DMFT - PUFA - Plaque score	X	X

*OHIP-14* Oral Health Impact Profile, *MDAS* Modified Dental Anxiety Scale, *BPI (SF)* Brief Pain Inventory (short form), *MOPDH* Modified Dental Anxiety Scale, *RESES* Rosenberg Self-Esteem Scale, *PHQ-9* Patient Health Questionnaire, *EQ-5D-5L* EuroQol 5 Dimension, *MINI* Mini International Neuropsychiatric Interview, *DMFT* = number of decayed, missing or filled teeth, *PUFA* Pulpal involvement, ulceration due to trauma, fistula, and abscess

We will access routinely collected dental visiting data alongside self-report. NHS England collects information on NHS dental visits and treatment through the NHS BSA. We will gather participants consent to access this data. We anticipate that the NHS BSA data will overcome problems around attrition and provide an objective measure of dental access. However, it only includes NHS, not private, dental visits and therefore may miss some appointments. We will establish the feasibility of using both self-report and NHS BSA data in this trial (see Table 1).

Outcome measures will include oral health quality of life using the Oral Health Impact Profile [37]; dental anxiety using the Modified Dental Anxiety Scale [38]; depression using the Patient Health Questionnaire [39]; orofacial pain using the Brief Pain Inventory Short Form [40]; pain related disability using the Manchester Orofacial Pain Disability Scale [41]; self-esteem using the Rosenberg Self-Esteem Scale [42]; and quality adjusted life years using the EuroQol 5 Dimension (EQ-5D-5L; [43]). We will also measure self-efficacy around dental visiting using the item ‘how confident are you that you will be able to attend a dental appointment?’ adapted from Armitage and colleagues [44]. Other outcomes include oral health self-hygiene behaviours, access to free/subsidised dental care, and the number of appointments attended. Participants will complete the Mini International Neuropsychiatric Interview [35] to assess mental health diagnosis at baseline to understand and present the diagnostic composition of the sample. We will also collect demographic and background clinical information on participants to allow for a detailed description of the mental health and oral health status of the sample. Senior investigators (JPC, CH, RoG) will provide training and supervision to the research assistants in the administration and scoring of the measures.

#### Dental examination

Participants will be offered an optional dental examination with a dental therapist alongside the other assessments to establish the condition of teeth and gums. The authors decided to make this optional to ensure that it did not prohibit participation in those people with severe mental illness who might find dental procedures most challenging. The dental therapist will use portable equipment and a headlamp to examine the oral cavity, following robust infection control procedures (e.g. latex gloves, sterilised instruments). This will take place in the same location as the other assessments. They will assess the number of decayed, missing and filled teeth (DMFT score); Pulpal involvement, ulceration due to trauma, fistula, and abscess (PUFA; [45]); and levels of plaque (modified plaque score; [46]). These measures are good indicators of oral health and frequently used in epidemiological surveys of oral health. Therapists will be trained, calibrated, and offered regular supervision by clinicians experienced in collecting clinical outcome data (VA, SP).

### Data collection

Research assistants will facilitate the self-report measures and semi-structured diagnostic interview. If possible, the dental therapist will complete the optional dental examination at the same appointment. Assessments will take place at the participant’s home, clinical base, doctors’ surgery, community hub, or third sector organisations. In the absence of the dental examination, we will also permit remote assessment via telephone or video call. This has previously proved useful when engaging participants who struggle to leave their accommodation [47]. If indicated, assessments may be completed over multiple appointments within 2 weeks of the date of consent. Follow-up assessments will be completed during a 4-week window commencing 9 months after the point of randomisation. Research assistants will offer updates and reminders about the study during the 9-month window via telephone, text, email, or letter to try to reduce attrition. Participants are free to withdraw from the intervention or trial at any time.

### Qualitative evaluation

The embedded qualitative study draws on the MRC evaluation of a complex intervention framework [48]. The key aims are: first, to understand the potential factors that may influence the acceptability and delivery of the intervention and a full RCT; second, to examine staff and participants’ experiences of the intervention with a focus on how it works within local health ecosystems. Up to twenty participants from the feasibility trial will be approached and consented to take part in an interview. Participants will be purposively sampled from across the three sites and both arms of the trial to capture the experiences of both the intervention and control arms.

**Fig. 1:**
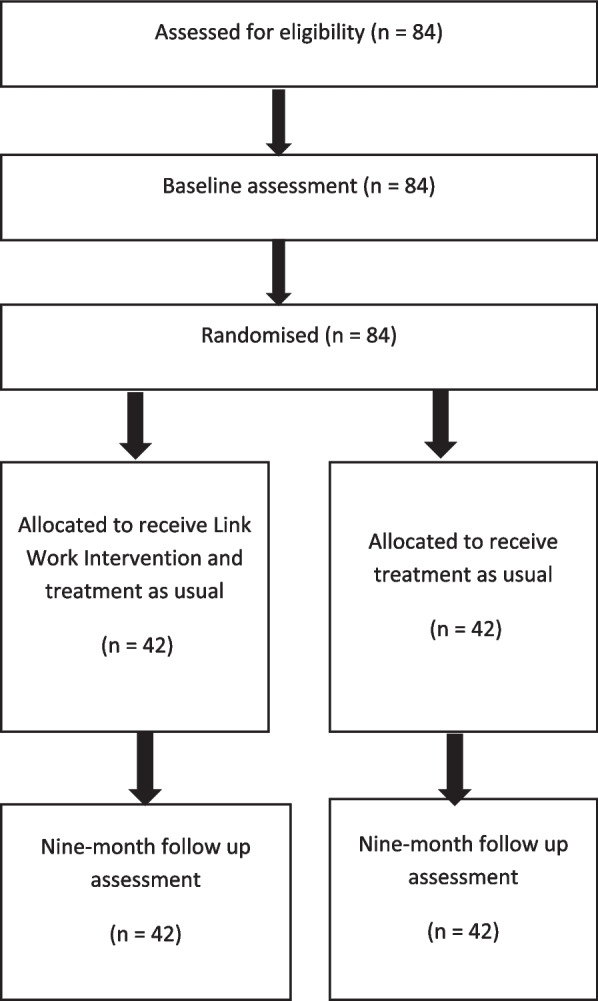
Flowchart for feasibility trial

Trial research assistants, link workers, and dental therapists will be invited to participate in interviews to investigate their experiences and insights of working on the trial. These interviews will be supplemented with data from reflective logs kept by staff. Relevant stakeholders (referrers to the study, service commissioners, and service managers) will also be approached to participate in interviews. A non-blinded researcher will conduct the interviews using topic guides developed through engaging with the existing literature and in collaboration with the PPI group. The interviews will be audio-recorded with permission and transcribed verbatim. If participants are not comfortable being audio-recorded, the interviewer will take written field notes. The data will be analysed iteratively and concurrently with data collection. This will allow arising topics to be explored in subsequent interviews and allow a dynamic approach to the study in which important findings can be used to iteratively inform the design of the feasibility trial. Thematic framework analysis using a hybrid deductive/inductive thematic approach will be used [49]. Thematic framework analysis is particularly useful for multi-disciplinary team working due to the structured nature of analysis (Fig. 1) [50].

### Trial oversight

The trial management group will bring together clinicians and academics specialising in severe mental illness and dentistry, trial statisticians, and people with lived experience. It will oversee the overall management of the project. There will be a site lead (PF, RG, JPC) at each of the participating NHS trusts overseeing localised recruitment and data collection. A trial steering committee (TSC) will meet biannually to offer independent guidance and oversight of the trial. This will consist of experts in mental health, dentistry, and trial delivery. PPI helped to shape the design of the trial protocol and intervention. A purposely convened PPI panel will meet quarterly throughout the project to offer ongoing support, advice, and feedback on the delivery of the trial. For example, they will provide feedback to research staff on how to deliver the assessments. The PPI panel will consist of people with lived experience of mental health difficulties and carers/family members. Meetings will be co-facilitated by a service user researcher (CL).

### Safety monitoring

We will closely monitor all adverse events and serious adverse events (SAE) occurring between baseline and the end of an individual’s participation in the trial. All trial staff will be required to report any SAE immediately and within 24 h using a set reporting template. The chair of the TSC (or in their absence the deputy chair) will independently review all SAE with a focus on their relatedness, expectedness, and severity. They can recommend pausing or stopping the research if there were any concerns about the safety of the trial procedures or intervention. We will state the number of SAEs in our final report.

### Sample size

The primary aim of this study is to test feasibility in terms of the recruitment rate, visiting data, and dental examination. Thus, there is no requirement for a power calculation based on assessment of efficacy. No previous studies have investigated a link work intervention in patients with severe mental illness and therefore no data exists on acceptability to the intervention. Eighty-four participants are to be recruited from three sites, 42 per randomisation arm. The sample size has been calculated to allow for 70% or more of expected participants to be recruited, thus the lower end of the 95% confidence interval will be above the 60% amber/red cut off point (see Table 1).

### Data management

The database has been developed in REDCap and it is held securely at the Liverpool Clinical Trials Centre. Data will be entered directly into the REDCap database at site by staff members trained in data entry by the trial team. The sites will enter all study data at the earliest possible opportunity. All study data will be stored separately to personal identifiable data and the only way of linking these will be via a participant identification number held by the research team. Personal data will not be shared outside of the study team except for auditing purposes and the NHS BSA application. Paper consent forms will be stored in a locked filing cabinet within a locked office on NHS premises. Audio consent recordings will be stored on secure NHS shared drives accessible only to members of the research team.

### Analysis

A detailed statistical analysis plan will be prepared prior to the collection of all data. All statistical analyses will be performed with standard statistical software (e.g. SAS version 9.4). Descriptive statistics will be presented as mean and standard deviations for continuous variables, and frequencies and percentages for categorical variables at baseline and follow-up, overall and by treatment group. All analyses will be according to the intention-to-treat approach, including all participants randomised, regardless of adherence to the study protocol.

The updated CONSORT 2010 Statement for randomised pilot and feasibility trials will be used to analyse outcome data at the end of the last follow-up assessment. Data on screening, willingness to be randomised, recruitment and loss to follow-up will be presented for each arm. The quantitative success criteria listed in Table 1 will be presented as numbers and percentages, with 95% confidence intervals. The efficacy of intervention will not be investigated, but analysis will include descriptive statistics of proposed outcome measures for the full trial, both overall and split by treatment group, which can be used to inform the design of a definitive trial.

### Ethics and auditing

The project is sponsored by Lancaster University and has received approval from an NHS research ethics committee (Wales Research Ethics Committee 2; ID: 304,696). Any changes to the approved protocol would require approval of an ethics amendment. The study may be audited by relevant agencies from the project sponsor or partners, but no external audits are currently planned. Liverpool Clinical Trials Centre will perform data checks via REDCap. The trial management team will monitor study protocol adherence and report major protocol deviations to the TSC, sponsor, and ethics committee.

### Dissemination

The authors will present the trial findings in academic journals and lay articles. They will present the findings at local and national events, including conferences and NHS research roadshows. The PPI panel will support the development of a lay infographic. The National Institute of Health Research will receive a full report of the findings. Authorship will be decided closer to the time of publishing.

## Discussion

People with severe mental illness experience poor oral health [2–4], but struggle to attend routine dental appointments [15], compared to the general population. The Mouth Matters in Mental Health Study will evaluate the feasibility and acceptability of a trial for a link work intervention that aims to bridge the gap between mental health and dental services. This feasibility RCT broader goal is to tackle the multiple barriers that people with severe mental illness face when attempting to access support and treatment for their teeth and gums. In the long term, this project aims to try to overcome the socioeconomic and practical barriers to help seeking within a model of socially engaged dentistry [51]. The research takes place within the current context of dentistry in the United Kingdom; the number of NHS dentists taking on new patients is limited and variable across regions [52], potentially widening existing inequalities around access. This may further necessitate, but pose challenges for, the link work intervention and requires careful exploration at the feasibility RCT stage.

The Mouth Matters in Mental Health Study is novel in that it will try to combine expertise and knowledge from the fields of dentistry and mental health. For example, dental measures and examinations have rarely been employed in people with severe mental illness within the context of an RCT [53]. Other RCTs attempting to improve the oral health of people with severe mental illness have focused on motivational interviewing, education, monetary incentives, dietary advice, and dental checklists [53, 54]. This trial will provide feasibility data on whether people receiving care around their mental health want and engage with a link work intervention that focuses on dental access. The RCT uses robust procedures for randomisation and blinding, whilst carefully monitoring levels of SAE to ensure the safety of its participants. PPI has helped to design the trial and intervention and will continue to steer the project’s delivery.

The Mouth Matters in Mental Health Study is due to finish in 2024. If the feasibility data shows promise, the authors will apply for funding to conduct the definitive trial to test effectiveness. Regardless of the findings of this study, there is major need to try to find evidence-based interventions and initiatives for improving the oral health of people accessing mental health services. Indeed, poor oral health should not be the inevitable consequence of experience severe mental illness.

### Supplementary Information

**Additional file 1. **SPIRIT 2013 Checklist: Recommended items to address in a clinical trial protocol and related documents*

## Data Availability

An anonymised final trial dataset will be available following publication of the study, at the discretion of the authors.
